# Supplementary material for: Splice-Junction-Based Mapping of Alternative Isoforms in the Human Proteome
Source: Cell Rep. Author manuscript; Available in PMC 2020 Jan 15. (PMC6961840; doi:10.1016/j.celrep.2019.11.026)
Supplement: 3 [file NIHMS1546469-supplement-3.zip › DF2/PXD009737/TestisGluC-29-P18206-ARKWSSKGNDIIAAAKR.pdf]

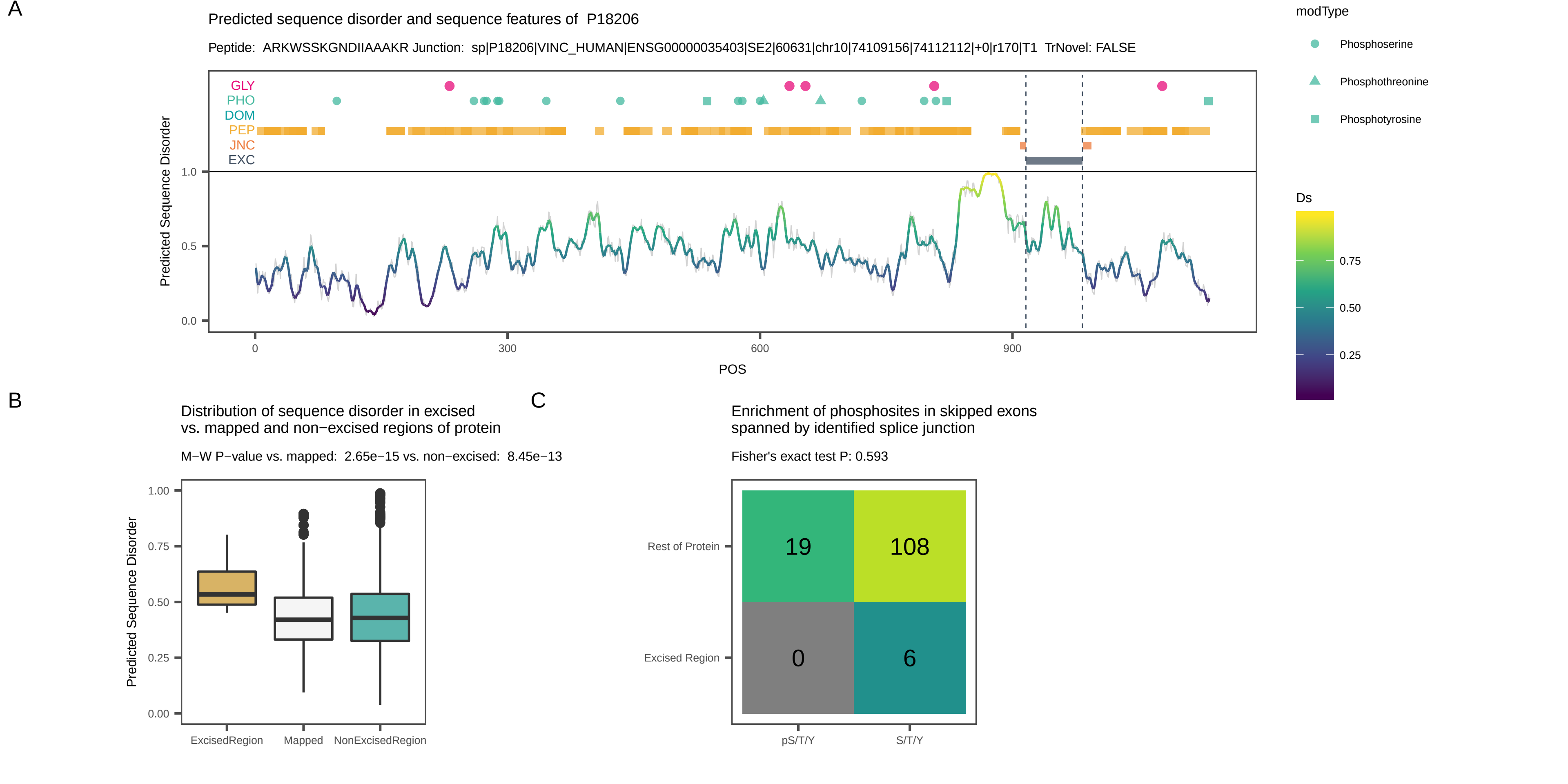
A
Predicted sequence disorder and sequence features of P18206
Peptide: ARKWSSKGNDIIAAAKR Junction: sp|P18206|VINC_HUMAN|ENSG00000035403|SE2|60631|chr10|74109156|74112112|+0|r170|T1 TrNovel: FALSE
GLY
PHO
DOM
PEP
JNC
EXC
Predicted Sequence Disorder
1.0
0.5
0.0
0
300
600
900
POS
modType
Phosphoserine
Phosphothreonine
Phosphotyrosine
Ds
0.75
0.50
0.25
B
Distribution of sequence disorder in excised
vs. mapped and non−excised regions of protein
M−W P−value vs. mapped: 2.65e−15 vs. non−excised: 8.45e−13
Predicted Sequence Disorder
1.00
0.75
0.50
0.25
0.00
ExcisedRegion
Mapped
NonExcisedRegion
C
Enrichment of phosphosites in skipped exons
spanned by identified splice junction
Fisher's exact test P: 0.593
Rest of Protein
Excised Region
19
108
0
6
pS/T/Y
S/T/Y
